# Cardiac and Vascular Target Organ Damage in Pediatric Hypertension

**DOI:** 10.3389/fped.2018.00148

**Published:** 2018-05-24

**Authors:** Michael Khoury, Elaine M. Urbina

**Affiliations:** Heart Institute, Cincinnati Children's Hospital Medical Center, Cincinnati, OH, United States

**Keywords:** echocardiography, vascular function, target organ damage, hypertension, left ventricular mass, cardiac function

## Abstract

Atherosclerosis begins in youth and is associated with the presence of numerous modifiable cardiovascular (CV) risk factors, including hypertension. Pediatric hypertension has increased in prevalence since the 1980s but has plateaued in recent years. Elevated blood pressure levels are associated with impairments to cardiac and vascular structure and both systolic and diastolic function. Blood pressure-related increases in left ventricular mass (LVM) and abnormalities in cardiac function are associated with hard CV events in adulthood. In addition to cardiac changes, key vascular changes occur in hypertensive youth and adults. These include thickening of the arteries, increased arterial stiffness, and decreased endothelial function. This review summarizes the epidemiologic burden of pediatric hypertension, its associations with target organ damage (TOD) of the cardiac and vascular systems, and the impact of these adverse CV changes on morbidity and mortality in adulthood.

## Introduction

Internationally, ischemic heart disease and stroke is the leading cause of death (1) and it is estimated that by 2030, over 40% of the US population will have cardiovascular disease (CVD) (2). Hypertension is an established modifiable cardiovascular (CV) risk factor, and by 2030, an estimated 41% of the US population will have hypertension (3). The incidence of CVD is associated in a linear manner with BP. A clear BP threshold at which the risk for CVD events occurs has not been identified (4). That said, prior to the development of overt clinical CVD, subclinical cardiac and vascular target organ damage (TOD) and atherosclerosis can be detected non-invasively using techniques such as measurement of left ventricular mass (LVM), carotid intima media thickness (cIMT), arterial stiffness, and endothelial function. These non-invasive techniques are being increasingly utilized in the pediatric population to identify early TOD associated with hypertension and other CV risk factors (5). In this review, we will provide an overview of the epidemiology of pediatric hypertension and its associations with cardiac and vascular TOD and adult sub-clinical and manifest CVD.

## Pediatric hypertension

### Prevalence of hypertension in children

Data from the National Health and Nutrition Examination Survey (NHANES) demonstrate that BP levels in the pediatric population rose from 1988 to 1999 (6) and have plateaued or even slightly decreased since then (7, 8). Increases in BP levels have paralleled the trend of increasing obesity prevalence (9). This is not surprising, as multiple studies have demonstrated associations between obesity and BP (10–13). The prevalence of increased BP levels, specifically elevated BP, (previously referred to as pre-hypertension) is lower in clinical studies where multiple measures of BP are obtained compared with epidemiologic studies. This is because BP levels naturally have a variability. In addition, the accommodation effect, whereby children adjust to the experience of having their BP taken, results in lower mean values. For example, recent NHANES data have estimated the prevalence of pediatric hypertension to be 1.6% and elevated BP to be 9.5% (8). Clinical studies, on the other hand, estimate a prevalence of hypertension at about 3.5% (12, 14), and the prevalence of elevated BP at about 2.2–3.5% (14–16).

Hypertension is more common in boys aged 8–17 than girls, and is more prevalent in African American and Hispanic children and in older children (8). The prevalence of hypertension is significantly higher in children with type 1 and 2 diabetes mellitus (17). For example, in the SEARCH for Diabetes study, a multi-center study of diabetes complications in youth, 24% of children and adolescents with type 2 and 6% with type 1 diabetes mellitus had hypertensive BP measurements (17). Pediatric hypertension is also more prevalent in children with various chronic conditions, including obesity, sleep apnea, chronic kidney disease, and a history of prematurity (15).

Routine measurement of BP beginning at age 3 at well-child visits is recommended to allow for the early detection of both primary and secondary hypertension (15). Despite this recommendation, elevated BP and hypertension can frequently go unnoticed in childhood and adulthood (3, 14, 15, 17). For example, in a study of children and adolescents 3–18 years of age who had undergone at least three BP assessments between 1999 and 2006, 3.6% had hypertension. Of these children, only about one-quarter had been previously diagnosed, whereas 74% had not been diagnosed. Elevated BP was found in about 3.5% of children, and only 11% of these children had been previously diagnosed (14).

Children and adolescents with elevated BP are at risk for progressing to sustained hypertension. A longitudinal assessment of 13–15 year olds from the National Childhood Blood Pressure database demonstrated that, of youth with elevated (pre-hypertensive) BP measurements initially, 14% of boys and 12% of girls developed sustained hypertension 2 years later. The rate of progression from elevated BP to hypertension was determined to be about 7% per year (13). Moreover, children with elevated BP are at risk of developing hypertension and metabolic syndrome in adulthood (18–23). In addition, a recent study by Kelly et al. demonstrated that children with elevated BP (pre-hypertension and hypertension) had a 35% increased risk of having elevated BP in adulthood compared to those with normal BP in childhood (24). Compared to children with sustained elevated BP into adulthood, those who's blood pressure normalized in adulthood demonstrated decreases in their BMI z-scores, decreases in alcohol consumption, and increases in vegetable intake. Therefore, the early identification of elevated blood pressure in childhood may allow for changes in lifestyle that may promote lifelong improvements in CV health.

### Definition of hypertension

A new Clinical Practice Guideline (CPG) was published by the American Academy of Pediatrics on the screening and management of elevated BP in children and adolescents in 2017 (15) and serves as an update to the previous hypertension guidelines published in 2004 (25). The CPG included an updated definition of pediatric hypertension (Table 1), and new reference tables for normative BP-values, removing youth with overweight and obesity due to the known association between overweight and obesity and BP (26, 27). This resulted in new reference tables with a 1–4 mmHg decrease in the thresholds for elevated BP (formerly termed pre-hypertension) and hypertension (28). The implications of such a change are that fewer children <13 years old with elevated BP measurements will be missed. The most prominent change, however, was the introduction of single BP cut-off values for adolescents >13 years old, as opposed to the normative-data, percentile-based definitions previously used. The cut-points proposed are the same as the recently updated adult hypertension guidelines (29) and are intended to simplify the process of identifying and classifying hypertension in adolescents and facilitate transition from care in adolescence to adulthood (15). In addition, the adult cut-points are based on hard CV outcomes (30) as opposed to the pediatric guidelines that traditionally had defined hypertension by percentiles from normative data that was not linked to events in adulthood (15, 25). While it has not been studied to date, the authors hypothesize that the new CPG may decrease the prevalence of systolic hypertension categorization in younger, shorter adolescents, and increase the prevalence of systolic hypertension in older adolescents (Table 2). The relatively conservative cut-point for diastolic hypertension (>80 mmHg) in the CPG suggests that the prevalence of diastolic hypertension classification likely increases through the use of the CPG (Table 2). This increased prevalence of hypertension may in turn improve the sensitivity of TOD detection in hypertensive patients due to the re-classification of patients with TOD from pre-hypertension by the Fourth Report to hypertension by the CPG. The earlier detection of increased LVM may have significant clinical implications as its presence may prompt clinicians toward earlier initiation of antihypertensive therapy, as supported by the CPG (15). A detailed overview of BP assessments and diagnosis and treatment of hypertension is found in the CPG (15).

**Table 1:** Blood pressure definitions, as defined by the 2017 Clinical Practice Guidelines (15).

**BP category**	Children aged 1–13 years^*^	**Children aged ≥13 years**
Normal BP	<90th percentile^†^	<120/<80 mmHg
Elevated BP	≥90th to <95th percentile	120/<80 to 129/<80 mmHg
Stage 1 HTN	≥95th to <95th percentile + 12 mmHg	130/80 to 139/89 mmHg
Stage 2 HTN	≥95th percentile + 12 mmHg	≥140/90 mmHg

* *If blood pressure values exceed criteria used for children aged ≥13 then those corresponding cutoffs are used*.

† *All percentiles are age-, sex-, and height-matched. Of note, the Clinical Practice Guidelines included new reference tables derived from only lean subjects*.

**Table 2:** A comparison of blood pressure threshold values for stage 1 hypertension in adolescents as defined by the Fourth Report and Clinical Practice Guideline (CPG).

	**Male**		**Female**	
**Age and height percentile**	**Fourth report**	**CPG**	**Fourth report**	**CPG**
**13 YEARS**				
5th percentile	121/79	130/80	121/80	130/80
50th percentile	126/81	130/80	124/81	130/80
95th percentile	130/83	130/80	128/83	130/80
**15 YEARS**				
5th percentile	126/81	130/80	124/82	130/80
50th percentile	131/83	130/80	127/83	130/80
95th percentile	135/85	130/80	131/85	130/80
**17 YEARS**				
5th percentile	131/84	130/80	125/82	130/80
50th percentile	136/87	130/80	129/84	130/80
95th percentile	140/89	130/80	132/86	130/80

## Non-invasive assessments of cardiac and vascular target organ damage

Atherosclerosis begins in childhood (31, 32), and autopsy studies have demonstrated evidence of increased atherosclerosis in youth with elevated BP levels (33, 34). Moreover, non-invasive assessments have demonstrated associations between BP in youth and the presence of sub-clinical atherosclerosis and adverse cardiac and vascular changes (35), including increased cIMT (36–38), arterial stiffness (39–41), and LVM both in childhood (35, 42–44) and adulthood (45). This is of key importance as non-invasive assessments of sub-clinical CVD, including LVM, cIMT assessments, and pulse wave velocity (PWV) measurements are associated with CVD events in adulthood (38, 41, 44). While echocardiographic evaluations for LVM in youth are recommended for those with stage 2 or persistent stage 1 hypertension (15), cIMT and PWV, in addition to other imaging and non-imaging vascular assessments, typically remain restricted for research use only. This is primarily because of a lack of normative data in the pediatric population. However, these tools are increasingly gaining use, given the importance of identifying early sub-clinical changes secondary to hypertension (5, 46).

### Echocardiography

Echocardiography is the most convenient and widely accepted tool for the evaluation of BP-related cardiac TOD in both adults (47) and children (15, 48). There are multiple ways to calculate LVM by echocardiography, including by M-mode, 2D measurements of the LV chamber and posterior wall dimensions in diastole, and more recently with 3D echocardiography (47). At our center, LVM is calculated from measurements obtained from M-mode images taken in M-mode. To control for changes in body size through adolescence, LVM should be indexed to body size. While some controversy remains regarding the best method (49), most pediatric specialists (44) index to height (ht)^2.7^. Although LVM >51 g/m^2.7^ predicts hard adulthood CV events (44), many pediatric specialists define elevated LVM in youth as ≥38.6g/m^2.7^ and reserve ≥51 g/m^2.7^ as an indication of significant LV hypertrophy (LVH) requiring more urgent treatment (48). In addition, percentiles of LVM index have been developed for younger children where normalization to ht^2.7^ is less linear across age (50).

LVM is positively associated with blood pressure (51) and LVH. Daniels et al. (52) have demonstrated that 8% of children with hypertension had an LVM above 51 g/m^2.7^, the adult cut-point associated with CVD outcomes (43, 52–56). Multiple other studies have confirmed this association (57–60). Unfortunately, even mild elevations in BP are associated with higher LVM compared to normotensive individuals, indicating that TOD may occur at levels well below the treatment thresholds currently recommended (35, 59). Non-Caucasians appear to be at increased risk for developing increased LVM at a given BP level (43, 61). The presence of obesity may complicate interpretation of LVM, since normotensive obese youth have higher LVM compared with hypertensive lean youth (62, 63). It is the obese, hypertensive youth that is at greatest risk of developing elevated LVM (56, 62). In our practice, echocardiography is performed to evaluate LVM in all patients with confirmed hypertension. We tend to recommend earlier initiation of antihypertensive therapy for patients with raised LVM (≥38.6 g/m^2.7^), as opposed to ongoing efforts to improve lifestyle habits (diet and exercise), as supported by the CPG (15).

**Figure 1:**
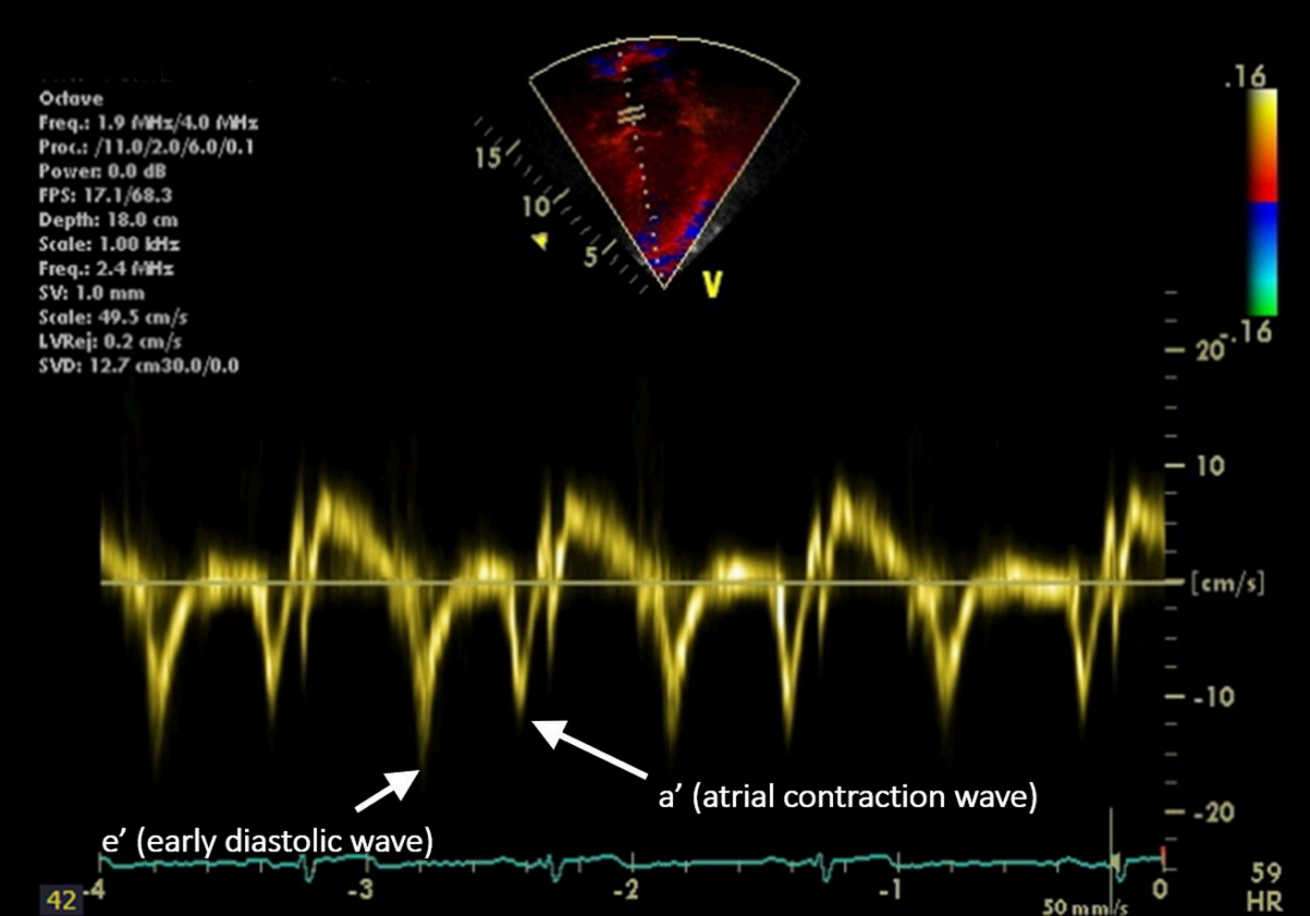
Longitudinal tissue Doppler imaging (TDI) obtained from an apical four-chamber view. The septal portion of the mitral valve is sampled. The peak early diastolic velocity (e′) and the peak late diastolic velocity (a′, representing atrial contraction) are demonstrated.

Measurement of cardiac function in hypertensive youth is also important since increased afterload and the development of vascular dysfunction (discussed below) results in increased LVM that may, over time, result in impaired diastolic and eventually, systolic function. The first measure of diastolic function developed was the ratio of the Doppler velocity of the early flow through the mitral valve “E-wave” to the velocity created by atrial contraction, the “A-wave” (E/A ratio) (64). This parameter is limited by its dependence on preload and afterload. Tissue Doppler Imaging (TDI), does not share this limitation. TDI evaluates the movement of the myocardium throughout the cardiac cycle (65) allowing for the evaluation of global diastolic function in addition to segmental analysis to evaluate regional differences in cardiac function (Figure 1). The E/A ratio the TDI e′ and a′ waves can be used together to calculate the E/e′ ratio which reflects LV filling pressure (65). Diastolic dysfunction as measured by TDI is an independent predictor of cardiac mortality in adults (66). Diastolic dysfunction has been associated with hypertension in both adults (67, 68) and the pediatric population (69). However, some studies have shown no association between BP and TDI measures of diastolic function (56, 70). Therefore, further evaluations of this potential relationship are needed.

**Figure 2:**
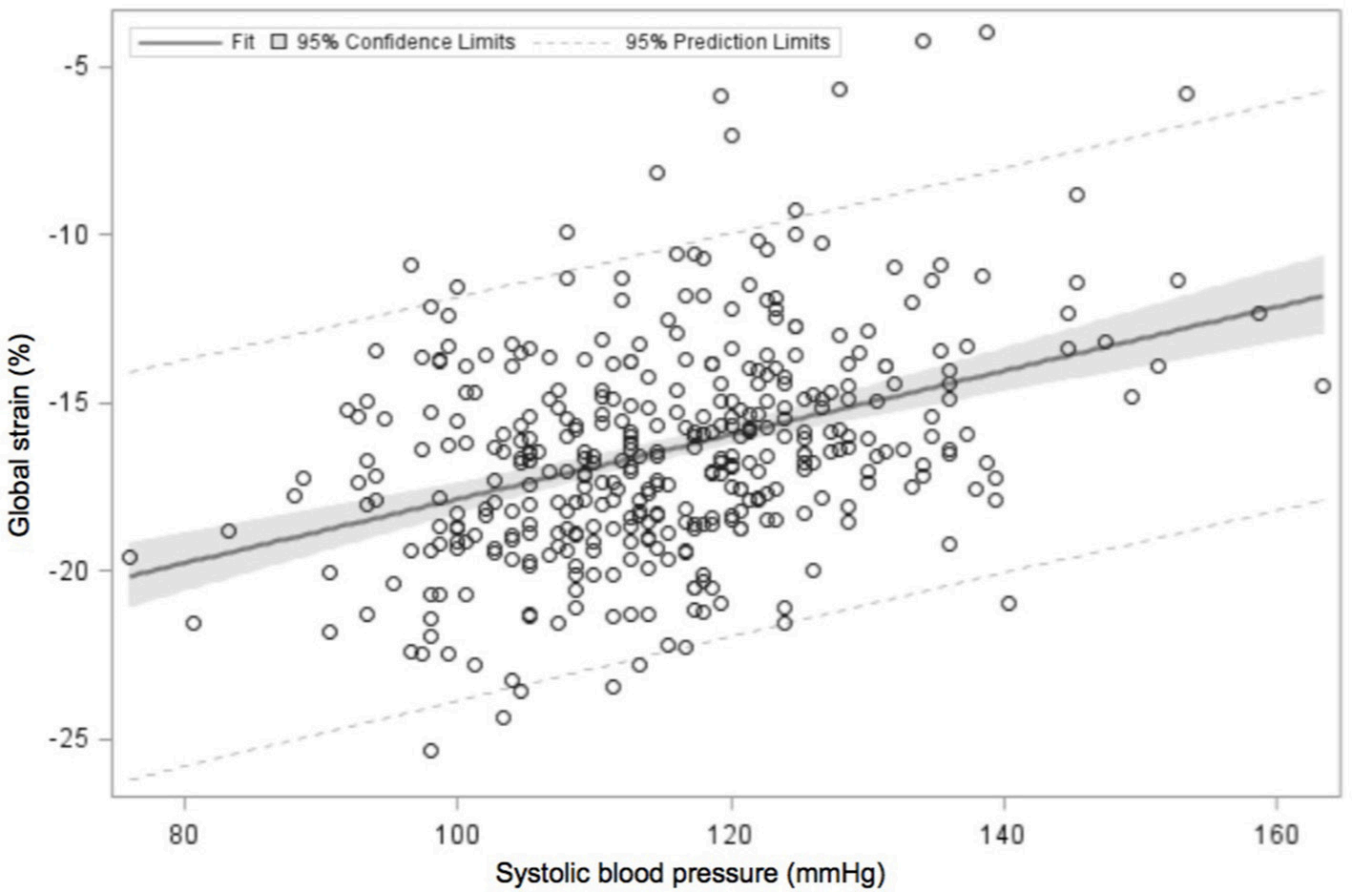
Linear regression analysis of global strain in the echocardiographic four-chamber view, plotted against systolic blood pressure. Data (unpublished to date) obtained from adolescents undergoing a study of the cardiac and vascular effects of obesity and type 2 diabetes mellitus. Global strain reduced with increasing systolic blood pressure values.

Systolic function is assessed via the shortening fraction (percentage by which the LV decreases in dimension during systole) or ejection fraction (percentage change in LV volume during systole) (64) or cardiac strain, which evaluates the changes in myocardial length, a measure of the deformation of the heart during the cardiac cycle. Strain rate, the speed of deformation, may be calculated using TDI or speckle tracking. Global longitudinal strain was as an independent predictor of death, even in adults with normal ejection fraction (71). Adults with hypertension have lower strain and strain rates compared with their normotensive counterparts (72, 73). A recent study demonstrated reduced strain (myocardial deformation) in adolescents with hypertension (74). This finding is in keeping with our observations to date, that global strain is inversely related to systolic blood pressure (Figure 2, Urbina, unpublished data).

Fortunately, antihypertensive treatment can reduce LVH in both children (75, 76) and adults (77–81) where regression in LVH is independently associated with improved CV outcomes (82). For patients with increased LVM, we typically re-assess LVM on an annual basis until normalization of LVM is noted. Studies in adults have demonstrated that treatment of hypertension results in significant improvements in diastolic function (83) and systolic strain (84). Improvement in cardiac function with treatment have also been seen in youth as increased shortening fraction was demonstrated in a pediatric patients with chronic kidney disease who underwent intensive BP treatment (85). Reduction in BP after weight-loss from bariatric surgery also yielded improvements in TDI measures (86), and an aerobic exercise intervention improved BP and cardiac strain in obese adolescents (87).

### Vascular testing

Vascular testing may include measures of vascular structure, arterial stiffness, or endothelial function. These tests are related but assess different properties of the arterial tree and may be affected by risk factors and treatments in different ways. Therefore, ideal assessments of a patient's vasculature involve a combination of techniques.

#### What is the importance of assessing vascular function?

“Hardening of the arteries” is a frequently employed lay term for atherosclerosis. The vascular changes associated with atherosclerosis eventually result in TOD and CV morbidity and mortality. Therefore, it is important to note that a direct relationship exists between sub-clinical measures of atherosclerosis such as increased cIMT (88) or increased arterial stiffness (89) and elevated LVM in youth. Findings such as these suggest that treatments aimed at improving vascular function in youth may prevent the development of CVD in adulthood.

#### Carotid ultrasound

cIMT is most commonly measured with “B-mode” ultrasound imaging of the common carotid, carotid bulb, and internal carotid artery at end diastole (5). While measurement of the femoral artery IMT is also possible, it has greater variability and strongly correlates with carotid measurements (90). Abdominal aortic thickness can also be measured but requires additional equipment, a fasting state, and may be less comfortable for younger patients (91). Therefore, we tend to limit our intima media thickness assessments to the carotid arteries.

Elevated cIMT is associated with CV events in adults (92) and pediatric BP levels as early as 9 years of age are associated with cIMT levels in young adulthood (93). Higher blood pressure in the pediatric population (94–97) including elevated but non-hypertensive BP levels have also been associated with thicker cIMT (35). Severity of BP elevation appears to be of importance, since cIMT was associated with higher daytime ambulatory systolic BP index (ratio of mean BP to the cut-point for hypertension) (98). Reduced nocturnal dipping is also associated with increased cIMT in diabetic youth (99). Associations have been demonstrated between cIMT and other CV risk factors in youth including hypercholesterolemia, obesity, diabetes, and metabolic syndrome (5, 37, 94). In fact, clustering of hypertension with other CV risk factors is associated with even thicker carotid arteries compared with those with hypertension alone (88, 100).

#### Arterial stiffness

Arterial stiffness is associated with CV events in adults (41, 101) and increased arterial stiffness may precede the development of hypertension (102). A number of techniques have been developed to assess the arterial wall, a system that behaves both as an elastic solid and a viscous liquid (103).

#### Ultrasound-based methods

During assessment of cIMT, M-mode (with its very high temporal resolution) of the common carotid artery is performed to obtain the maximal (during systole) and minimal (during diastole) diameters to calculate carotid stiffness (5). Increased carotid stiffness has been found in both pre-hypertensive (35) and hypertensive (104) adolescents and in youth with elevated BP due to chronic kidney disease (105), and post renal transplantation (106). Moreover, data from the Amsterdam Growth and Health Longitudinal Study demonstrated that higher BP levels and steeper increases in BP in youth were associated with increased carotid stiffness in adults aged 36 years (107).

#### Non-ultrasound-based methods

Non-ultrasound-based methods for evaluating arterial stiffness have also been developed. One such method, brachial dispensability (BrachD), involves the use of a cuff-based BP device. Similar to carotid stiffness, BrachD is lower (stiffer vessel) in youth with pre- or sustained hypertension (35) and in youth with obesity and type 1 and 2 diabetes mellitus (39, 108).

**Figure 3:**
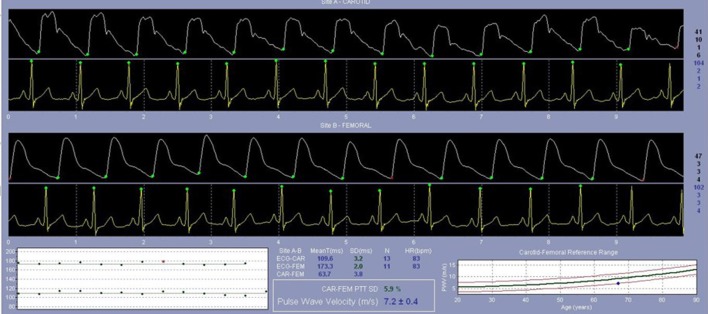
Femoral artery pulse wave velocity (PWV) assessment. Directly following an assessment of the carotid artery, an ECG-gated assessment of the PWV at the femoral artery is performed to evaluate the speed by which blood moves along the arterial tree.

**Figure 4:**
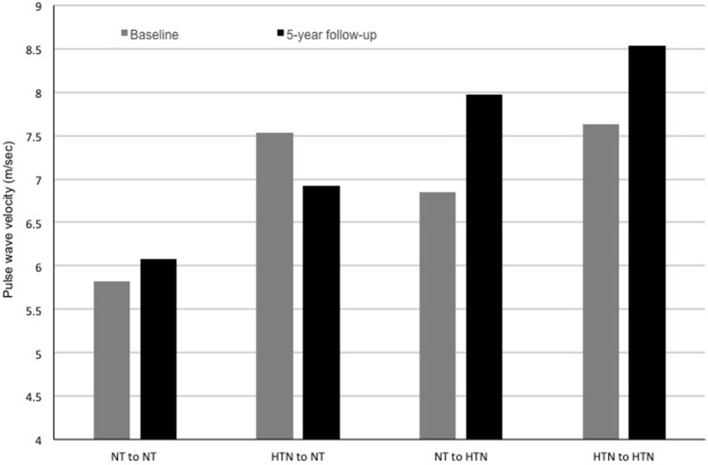
Pulse wave velocity (PWV) at baseline and at a 5-year follow-up in a population of adolescents undergoing a study of the cardiac and vascular effects of obesity and type 2 diabetes mellitus (data unpublished to date). Participants are categorized based on their blood pressure category (normotensive or hypertensive) at baseline and at follow-up. Participants who were hypertensive at baseline and normotensive at follow-up had an improvement in PWV. Normotensive participants who were hypertensive at follow-up had increased PWV. Participants who were hypertensive at baseline and at follow-up had increased PWV at both time points with evidence of a progression of PWV at the follow-up. NT, normotensive; HTN, hypertension.

PWV is the speed by which blood moves along the arterial tree. It is typically measured from an ECG-gated arterial pulse at a proximal (carotid) and distal artery (typically femoral) using a pressure tonometer allowing for determination of the pulse transit time (Figure 3) (5). PWV predicts hard CV events in adults even after adjusting for concurrent levels of CV risk factors (41). Studies show that adolescents with pre-hypertension have higher PWV (stiffer arteries), independent of obesity status (40, 35, 109). Patients with successfully repaired coarctation of the aorta also have a higher PWV if they have hypertension detected via ambulatory monitoring (110) or if they demonstrated an exaggerated response during exercise testing (111). PWV was also related to higher BP levels in patients post kidney (112) or heart transplantation (113), and in those with systemic lupus erythematosus (114). Multiple other studies have demonstrated associations between PWV and other CV risk factors, including insulin resistance (115), type 1 (108), and type 2 diabetes mellitus (39), reduced physical activity (116), and chronic kidney disease (117). PWV in adolescents appears to worsen as those teens enter a higher BP category at a 5-year follow-up and improve as they enter a lower BP category (Figure 4, Urbina, unpublished data). PWV measurements are the method of choice for assessments of arterial stiffness in most contemporary vascular studies.

Augmentation index (AIx) is an assessment of arterial stiffness that involves assessing wave reflections through tonometry. As blood moves through the arterial system, each pulse comes in contact with various branch points in the body, allowing for wave reflections back to the heart. The cardiac afterload to the heart is therefore a summation of the outgoing and reflected waves (118). AIx is the difference in pressure between the main outgoing pulse wave and the incoming reflected waves. It is displayed as a percentage of the pulse pressure that is normalized to a heart rate of 75 bpm. Stiffer arteries reflect the returning wave more quickly which arrives earlier in systole, thus “augmenting” the central aortic pulse pressure (cardiac afterload) and reducing coronary perfusion which occurs during diastole (103). This increase in demand with decreased supply explains the observation that a 10% increase in AIx results in an increased risk for CV events of (relative risk 1.318, 95% CI 1.093–1.588) (119). Increased AIx has been noted in hypertensive youth (35), and in those with obesity-related insulin resistance (115), T2DM (39), low physical activity levels (116), and CKD (118).

Arterial stiffness can also be calculated from 24-h ambulatory BP recordings. The ambulatory arterial stiffness index (AASI) is calculated as 1 minus the regression of the 24-h systolic on diastolic BP readings. As it is dependent on both cardiac output and arterial function (pulse pressure), it may serve as an indicator of ventricular-arterial coupling (120). Given this dependence, however it is not a “pure” measure of arterial stiffness as it is influenced by factors other than vascular function (121). AASI has been shown to be elevated in children and young adults with both primary hypertension and in those with repaired coarctation of the aorta (122). It has also been shown to be elevated in children with type 1 diabetes with hypertension, compared to both non-diabetic controls and non-hypertensive diabetic patients (123).

#### Endothelial function

In contrast to arterial stiffness which measures intrinsic arterial wall function, endothelial function evaluates the response of the endothelium to a stress (103). Currently, the gold standard of assessing endothelial function is the ultrasound-based measurement of brachial flow-mediated dilation (FMD). After occlusion of the brachial artery for 5 min with an inflated BP cuff, the percentage increase in diameter of the brachial artery is calculated with greater dilation indicating higher endothelial reactivity. Brachial FMD is challenging to measure in a reproducible fashion, and requires ongoing training and rigorous quality controls. Despite these limitations, adults with metabolic syndrome who have reduced FMD have been shown to be at a greater risk for future CV events (124). Since FMD studies may be difficult to perform in younger children due to the discomfort associated with continuous inflation of a BP cuff on the forearm for 5 min (5), pediatric data are limited. However, FMD was found to be reduced in children with renal impairment, compared with age-matched controls (125, 126) and Aggoun et al. (127) found FMD was inversely associated with mean ambulatory BP in pre-pubertal children.

A non-ultrasound assessment of endothelial function is provided by the EndoPAT (Itamar Medical, Caesarea, Israel) device. A small cuff is placed on one finger of each hand and the reactive hyperemic index (percentage increase in blood flow) is measured following an ischemic stimulus from an inflated cuff above the patient's systolic BP for 5 min (128).

Laser Flow Doppler (LFD) may be appropriate for evaluating endothelial function in younger children as one may avoid an ischemic insult by using a heating stimulus on the forearm to evaluate the microvascular. Adults with hypertension have been shown to have a reduced response to localized heating (129). While LFD has not been well studied in the pediatric hypertension population, a reduced response has been noted in youth with type 1 diabetes (130) and in diabetic youth with elevated BP (131).

Venous plethysmography uses a strap placed around the mid forearm and inflatable BP cuffs on the upper arms and wrist to allow arterial inflow without venous outflow. A stimulus (often local infusion of vasoactive substances) is applied and the change in limb circumference is measured by a strain gauge. By confining the study to a limb, plethysmography avoids counter-regulatory systems such as increases in heart rate that may occur with vasodilation. Using this technique, endothelial function was lower in obese 10 year-old children with handgrip and mental stress testing compared with their lean counterparts (132).

#### How can vascular function be improved?

Primordial prevention, or the prevention of acquisition of CV risk factors is the first step for preventing accelerated vascular aging. Data from the Bogalusa Heart Study (133) and the Young Finns Study (134) demonstrated that lower levels of CV risk factors in childhood was associated with decreased cIMT in adulthood. Furthermore, if childhood metabolic syndrome had resolved by adulthood, the cIMT in those individuals was not different from those with low levels of risk factors throughout the lifespan (135). The Young Finns study has also shown that lifelong increased fruit and vegetable consumption is associated with lower PWV as an adult (136). In youth with type 1 diabetes mellitus, possessing a greater number of ideal CV health metrics was associated with reduced PWV and AIx (137). Improvements in vascular parameters have also been noted with primary prevention in youth (treatment of existing risk factors) including lower BP and cIMT in children after weight loss (138), and improved BP and forearm blood flow (endothelial function) in youth undergoing a diet and exercise program (132).

Secondary prevention (aggressive treatment in high risk youth) also results in improvement in vascular function including slower progression of cIMT in youth with pediatric familial hypercholesterolemia treated with statins (139, 140). In pediatric patients with chronic kidney disease, both carotid thickness (141) and stiffness (126) demonstrate improvements following kidney transplantation. Removal of tonsils and adenoids for obstructive sleep apnea also improves BP and LFD response (142). Unfortunately, no randomized clinical trials evaluating the vascular response to the initiation of anti-hypertensive medications have been performed to date.

### Future directions

Before vascular assessment can be used clinically, normative data are required across age, body size, and race/ethnicity. Although some epidemiologic studies using PWV have included some healthy children (143–146), and children with hypertension (35), obesity, and diabetes (39), there is a lack of consistency in techniques used across these studies, so normal values can only be interpreted for the device used and the population studied. As such, the lack of standardization for the techniques and measurements involved in vascular assessments is a key limitation preventing their clinical application. In 2009, a Scientific Statement was published by the AHA providing a standardized approach to vascular assessments in youth (5). The implementation of such standardized protocols may allow for the generation of normal values that in turn would be a large step toward incorporation into clinical practice. In addition, longitudinal data are lacking throughout childhood and adolescence. Finally, larger interventional trials are needed to prove which therapies are effective in reversing risk factor-related CV decline in youth.

## Conclusions

Hypertension is a prevalent CV risk factor in children and adults and is associated with increased vascular thickness and stiffness, reduced endothelial function, and adverse changes to cardiac structure and function. These changes are strongly associated with the occurrence of hard CVD end-points in adulthood. Fortunately, lifestyle and pharmacologic intervention studies have demonstrated improvements in vascular and cardiac health in both hypertensive patients and those with other CV risk factors. However, much more work remains to be done, particularly with respect to normative data for vascular assessments in the pediatric population and evaluations of treatments to improve CV structure and function in an effort to reduce the significant burden of CV diseases around the world.

## Author contributions

MK and EU made substantial contributions to the conception or design of the work, drafting the work and revising it critically for important intellectual content. MK and EU provided approval for publication of the content and agrees to be accountable for all aspects of the work in ensuring that questions related to the accuracy or integrity of any part of the work are appropriately investigated and resolved.

### Conflict of interest statement

The authors declare that the research was conducted in the absence of any commercial or financial relationships that could be construed as a potential conflict of interest.
